# Mediators for the Effect of Compassion Cultivating Training: A Longitudinal Path Analysis in a Randomized Controlled Trial Among Caregivers of People With Mental Illness

**DOI:** 10.3389/fpsyt.2021.761806

**Published:** 2021-12-07

**Authors:** Nanja Holland Hansen, Lone Overby Fjorback, Morten Frydenberg, Lise Juul

**Affiliations:** ^1^Department of Clinical Medicine, Danish Center for Mindfulness, Aarhus University, Aarhus, Denmark; ^2^MFStat, Aarhus, Denmark

**Keywords:** intervention, compassion, mediator, caregivers, mental health, depression, anxiety, stress

## Abstract

**Background:** There is a paucity of research on mediators of change, within compassion training programs. The aim was to investigate the mediators, of an 8-week compassion cultivation training (CCT) program, on the effect of psychological distress on caregivers of people with a mental illness.

**Method:** Longitudinal path models in a randomized controlled trial (RCT). One hundred ninety-two participants were assessed for eligibility, and 161 participants were included into the trial and randomized. The main outcome was psychological distress measured by the Depression, Anxiety and Stress scale at 6 months. Mediators included self-compassion (SC), mindfulness (FM), emotion regulation (ER), emotion suppression (ES), and cognitive reappraisal (CR). Baseline, post, and 3- and 6-month follow-up measurements were collected.

**Results:** The mediated effects for CCT are as follows: depression at 6 months: SC: −1.81 (95% CI: −3.31 to −0.31); FM: −1.98 (95% CI: −3.65 to −0.33); ER: −0.14 (95% CI: −1.31 to 1.02); anxiety at 6 months: SC: −0.71 (95% CI: −1.82 to 0.40); FM: −1.24 (95% CI: −2.39 to −0.09); ER: 0.18 (95% CI: −1.04 to 1.40); stress at 6 months: SC: −1.44 (95% CI: −2.84 to −0.05); FM: −2.17 (95% CI: −3.63 to −0.71); ER: −0.27 (95% CI: −1.51 to 0.98).

**Conclusion:** Mindfulness and self-compassion are important components in reducing psychological distress experienced by informal caregivers of people with a mental illness. Results contribute to the knowledge about the underlying mechanisms of CCT.

## Introduction

Poor mental health is on the rise (1), and there is a need for evidence-based interventions that decrease psychological distress and increase overall wellbeing. Compassion-based training programs may be one way to address this need (2). Compassion can be understood as the willingness to feel the suffering of oneself and others and to do something to relieve the suffering (3). Compassion is essential in caregiving and can become a practice of turning toward suffering rather than away and is a trainable skill (4).

Despite growing evidence, the processes underlying treatment response remain unclear (5). In fact, there is a paucity of research on the mediators (an intervening variable that may statistically account for the relationship between the independent and dependent variables) of compassion training programs (6, 7). To our knowledge, only one randomized controlled trial (RCT) has investigated the mediating variables of a self-help book intervention that was based on the principles of a compassion-based therapy (7, 8). Results showed that positive affect significantly mediated wellbeing and depressive symptoms, and negative affect significantly mediated wellbeing and anxiety symptoms (7).

Four RCTs investigated mediators of an 8-week Mindfulness-Based Stress Reduction (MBSR) program on anxiety disorders (6). Results suggested that the effects of the MBSR program were mediated by increases in positive self-views, decentering, and mindfulness. Limitations included small sample size and only two time-points of measurement (6). Systematic reviews (6, 9, 10) investigating the mechanisms of Mindfulness-Based Interventions (MBIs) found evidence supporting the mediating role of mindfulness and compassion (9), moderate evidence for mindfulness, and insufficient evidence for self-compassion and psychological flexibility (10). Moreover, greater self-reported changes in mindfulness lead to greater mediated clinical outcomes (6). An important limitation of all the studies included were that none of them fully met Kazdin's criteria for examining treatment mechanisms (11), namely, (1) a clear association between change in the proposed mediator and the proposed outcome and (2) that change in the mediator precedes change in the outcome (11). The studies included some theory, wide variability in measures used, time assessments being not optimal to test mediators, and all studies failing to assess whether changes in the mediators preceded the changes in the outcomes (6).

We recently published a trial showing effect on mental health of a compassion cultivation training (CCT) program in informal caregivers of people with a mental illness (12). The RCT showed the effect of CCT on outcomes as well as proposed mediators: mindfulness (FM), self-compassion (SC) emotion regulation measures (ER), cognitive reappraisal (CR), and emotion suppression (ES) (12). However, whether it was the effect on the mediators that led to the effect in the outcomes is unknown and requires further investigation.

Therefore, the expected link between intervention activities, mediators, and outcomes can be depicted by use of a logic model (13, 14). It has been suggested to divide a logic model into two: an action theory and a conceptual theory (15). The conceptual theory describes how the mediators are related to the outcome(s). The action theory describes how the intervention is supposed to affect the mediators. Our effectiveness trial supported the action theory as it showed statistically significant effects of CCT on mindfulness, self-compassion, and emotion regulation (12). Our predefined conceptual theory was based on the Process Model of Emotion Regulation developed by Gross and John (16). Numerous studies have shown that cognitive reappraisal and expressive suppression are among the most common explicit cognitive emotion regulation strategies (17) and are considered to have an important impact and effect on mental health and overall wellbeing. Difficulties in emotion regulation are related to psychological problems, contributing to depression and anxiety (17).

We therefore hypothesized that training in mindfulness allowed for informal caregivers to become aware of what they were currently experiencing (18), and training in self-compassion allowed participants to turn toward their own suffering in a kind and caring manner (19). This in turn allowed caregivers to increase cognitive reappraisal and decrease expressive suppression (17). Specifically, an increase in scores of self-compassion and mindfulness will mediate the effects of emotion regulation skills, i.e., increase in cognitive reappraisal and decrease in expressive suppression, which will mediate the effects of psychological distress in informal caregivers. The aim of the current study was therefore to estimate the mediating effects of mindfulness, self-compassion, and emotion regulation for CCT on symptoms of depression, anxiety, and stress at 6 months of follow-up, in caregivers of people suffering from a mental illness.

## Methods

### Study Design and Participants

The current study design is a longitudinal path model in an RCT. This is a secondary mediation analysis of a published RCT (12) comparing a CCT intervention to a waitlist control group for informal caregivers of people with a mental illness. Details of the RCT and adherence to the Consolidated Standards of Reporting Trials (CONSORT) reporting guidelines have been reported previously (12). The trial was conducted in two different community settings in Denmark, and ethical approval was obtained at the Central Denmark Region Committee of Health Research Ethics (De Videnskabsetiske Komitéer for Region Midtjylland) with approval 238/2017. The study was registered in ClinicalTrials.gov NCT03730155 before commencement. One-hundred and sixty-one caregivers of a relative with a mental disorder were included in the study.

A computer algorithm with predefined concealed random numbers was used for the block randomization with 40 participants in each block all randomized at the same time. To be included in the RCT, participants had to be an informal caregiver [e.g., a parent/spouse/adult child/sibling of a person suffering from a mental illness; all mental illnesses were included as described in Diagnostic and Statistical Manual of Mental Disorders−5 (DSM-5)] (20), between the age of 18 and 75 years and Danish speaking. Participants were excluded if they had a diagnosed and untreated mental illness, if they suffered from addictions, if they had a meditation practice, or if they received current psychotherapeutic treatment.

Data were collected between May 2018 and March 2019. There were nineteen males and 142 females, with a mean age of 52.6. *N* = 79 were randomized into the intervention group, and *N* = 82 were randomized into the waitlist control group [see Hansen et al. (12) for more detail]. Demographic measures were similar for both groups at baseline (Table 1). Measures were completed at four time points: baseline and at the 2-, 3-, and 6-month follow-up (12).

**Table 1:** Demographic characteristics of caregivers at baseline.

	**Intervention**	**Control**	**Total**
	***N*** **= 79**	***N*** **= 82**	***N*** **= 161**
Gender (n/%)			
Male	11 (14.1)	8 (9.7)	19 (11.8)
Female	68 (85.9)	74 (90.2)	142 (88.2)
Age (mean, sd)	55.9 (13.3)	49.5 (10.8)	52.6 (12.5)
Educational level (n/%)			
No high school	1 (1.3)	1 (1.2)	2 (1.2)
High school	4 (5.1)	2 (2.4)	6 (3.7)
Trade school	5 (6.3)	10 (12.2)	15 (9.2)
Short continuing education	8 (10.1)	3 (3.7)	11 (6.8)
Medium continuing education	43 (54.4)	25 (30.5)	68 (42.0)
Long continuing education	17 (21.5)	38 (46.3)	55 (34.0)
Ph.D.	0 (1.3)	1 (3.7)	4 (2.5)
Other	1 (0.0)	0 (0.0)	1 (0.6)
Years of caretaking (n/%)			
0–5	22 (28.2)	22 (27.1)	45 (28.1)
5–10	23 (29.5)	20 (24.7)	43 (26.9)
10–15	5 (6.5)	16 (19.8)	21 (13.1)
15–20	9 (11.5)	5 (6.2)	14 (8.8)
>more than 20	19 (24.4)	18 (22.2)	37 (23.1)
Patient psychiatric disorders			
Anxiety	18 (22.2)	35 (42.7)	53 (32.7)
ADHD	10 (12.7)	14 (17.1)	24 (14.8)
Autism	17 (21.5)	14 (17.1)	32 (19.8)
Bipolar disorder	9 (11.4)	12 (14.6)	21 (13.0)
OCD	6 (7.6)	12 (14.6)	18 (11.1)
Depression	19 (24.1)	21 (25.6)	40 (24.7)
Addiction	10 (12.7)	8 (9.8)	18 (11.1)
Personality disorders	8 (10.1)	13 (15.9)	21 (13.0)
PTSD	7 (8.7)	7 (8.5)	14 (8.6)
Schizophrenia	21 (26.6)	13 (15.9)	34 (21.0)
Eating Disorder	7 (8.9)	3 (3.7)	10 (6.2)
Stress	6 (7.6)	9 (11.0)	16 (9.9)
Acquired brain injury	6 (7.6)	6 (7.3)	12 (7.4)
Other	7 (8.7)	10 (12.2)	17 (10.5)
Mediators at baseline (N, Mean, SD)			
SCS (C)	76 36.45 (7.20)	80 34.9 (8.13)	
FFMQ (F)	79 37.32 (5.96)	77 37.31 (7.37)	
ERQ (E-R)	79 25.57 (7.23)	81 24.54 (6.71)	
ERQ (E-S)	79 12.68 (5.18)	80 12.1 (4.34)	

*Other included: disruptive behavior, bodily distress syndrome (BDS), mental retardation, psychogenic non-epileptic seizures (PNES), Parkinson, schizotypal, attachment disorder, Tourette, dementia*.

*Caregivers often had loved ones with comorbid disorders; therefore, the numbers do not total 100*.

*SCS, Self-Compassion Scale-12; FFMQ-15, Five-Facet Mindfulness Questionnaire-15; ERQ, Emotion Regulation Questionnaire*.

### Intervention

CCT is an 8-week manualized compassion training program, developed in 2009 at Stanford University (5, 21). The program has a dual focus on training compassion and loving kindness for one's own suffering and the suffering of others, and an implicit focus on mindfulness [for a more detailed description of the program format, please see Hansen et al. (12)]. Participants meet weekly for 2 h, and the group intervention was delivered face-to-face with 20 participants per group.

### Blinding

Owing to the nature of the intervention, the participants and the CCT instructor were aware of the treatment allocation and the researchers (NHH and LJ) were not blinded to group assignment when analyzing the data.

### Outcome and Mediator Measures

We assessed the primary outcome of psychological distress using the 42-item Depression Anxiety Stress Scale (DASS), which measures symptoms of depression, anxiety, and stress (22). The internal consistency for each of the subscales is high: Depression scale, Cronbach's α of 0.91, Anxiety scale 0.84, and Stress scale 0.90 (22). We assessed the mediators using the 12-item Self-Compassion Scale (SCS) (23), designed to measure participants' level of self-compassion with a correlation *r* = 0.97, which is very high between the SCS-26 and the SCS-12 for a total score of self-compassion. The 15-item Five Facet Mindfulness Questionnaire (FFMQ-15) (24) is designed to measure five facets of mindfulness, and the internal consistency has been found to be adequate for the FFMQ-15 (25). Lastly is the 10-item Emotion Regulation Questionnaire (ERQ), which measures two different emotion regulation strategies—cognitive reappraisal (ER) and expressive suppression (ES) (16). ERQ cognitive reappraisal (ER) (α = 0.89–0.90) and expressive suppression (ES) (α = 0.76–0.80) scores showed between acceptable to excellent levels of internal consistency and reliability (16). Danish versions of the instruments were used. The covariates were sex, age, educational level, years as informal caretaker, schizophrenia, and anxiety (diagnosis of loved ones).

### Statistical Analysis

**Figure 1:**
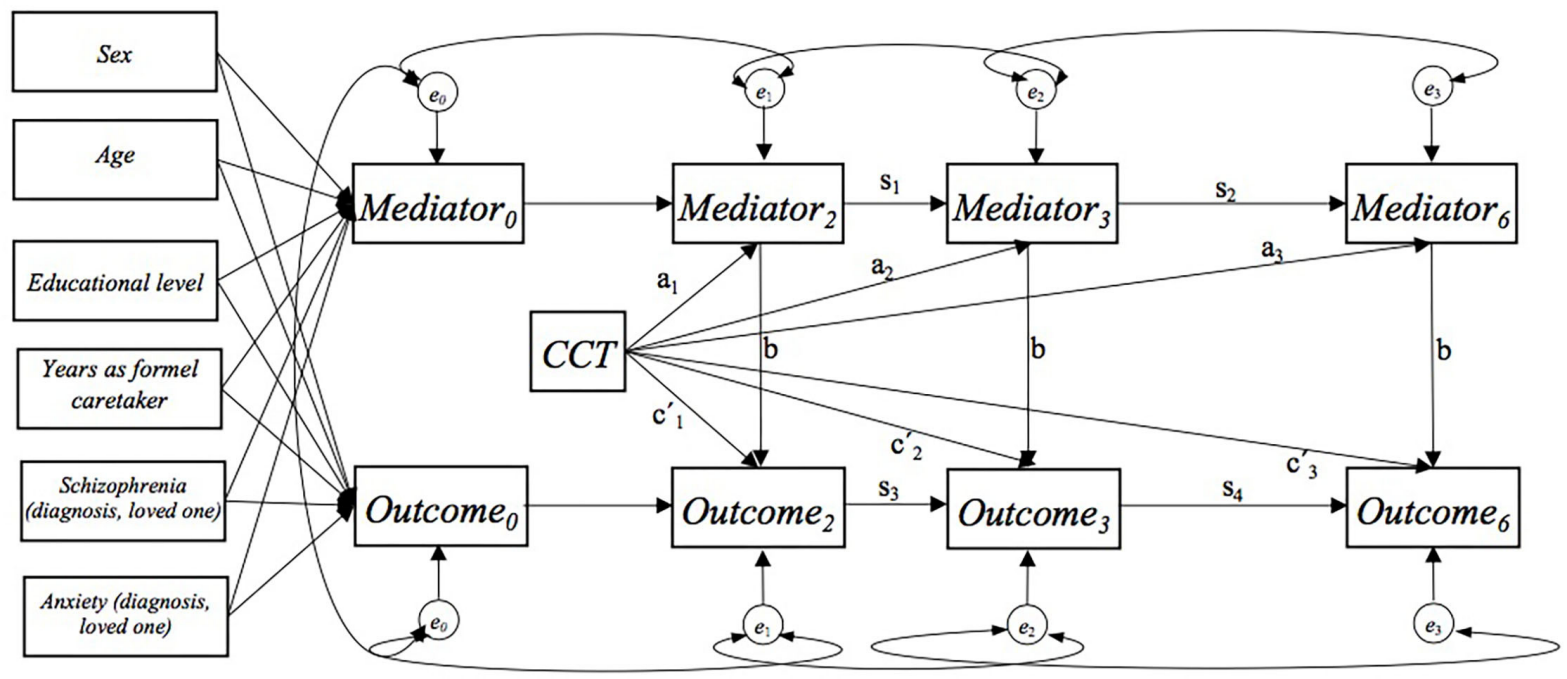
Autoregressive model with a single mediator showing the a, b, and c′ paths. At baseline, residual covariance between all mediators and outcome. For each mediator and the outcome residual covariance over time.

**Figure 2:**
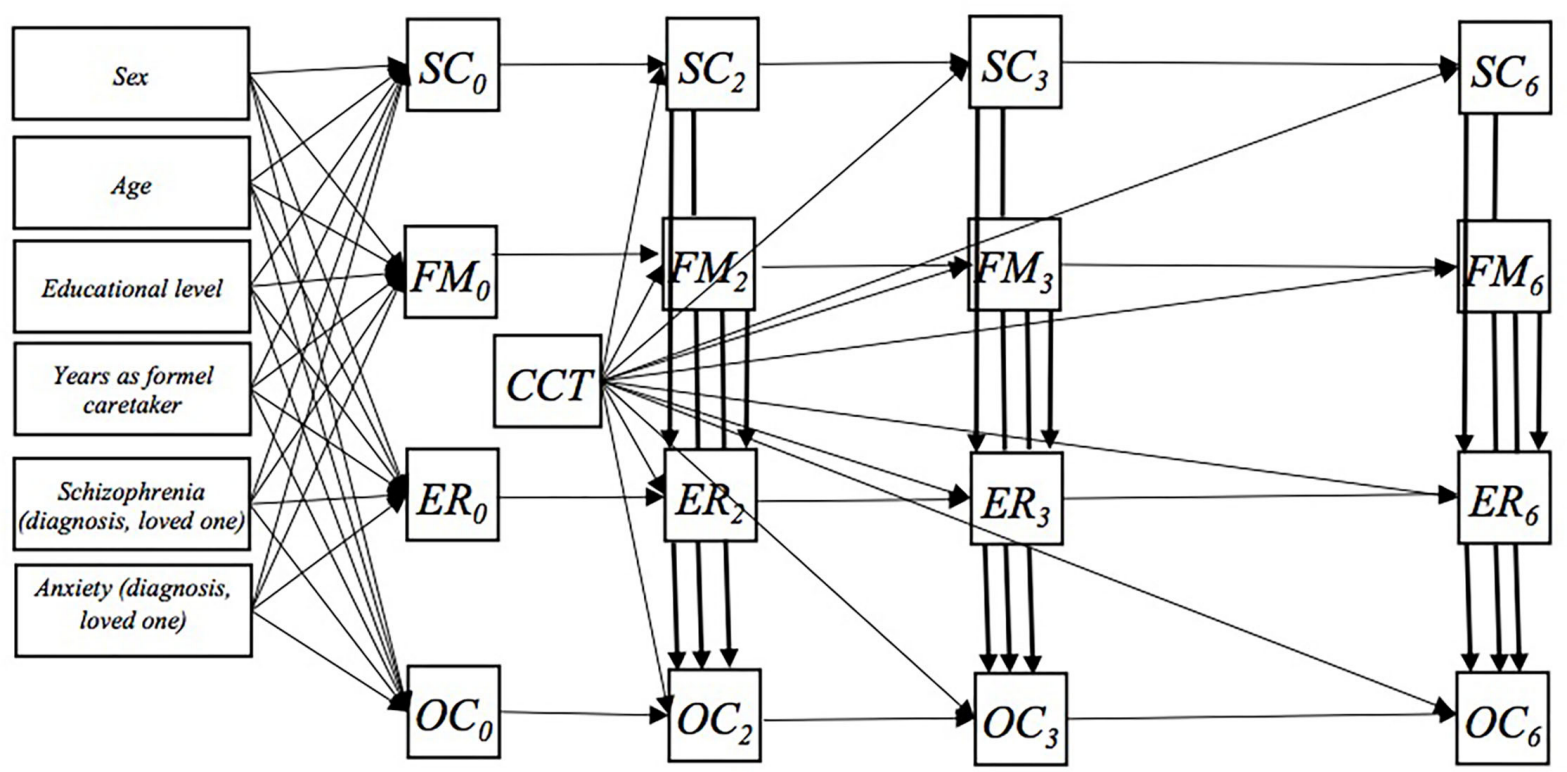
The paths in the main model including three mediators (SC, Self-Compassion Scale-12; FFMQ-15, Five-Facet Mindfulness Questionnaire-15; and Cognitive Reappraisal; ER, The Emotion Regulation Questionnaire. Not shown in the figure: At baseline, residual covariance between all mediators and outcome, OC. At each follow-up time, residual covariance between all mediators. For each mediator and the outcome residual covariance over time.

We analyzed the data using autoregressive models, with four time points of measurement and contemporaneous and constant b paths (26, 27) (Figure 1). The data were checked for normal distribution prior to analysis. For each outcome, we analyzed four models that included each mediator as a single mediator. For each outcome, we analyzed a model that included all mediator variables, which had shown to be statistically significant mediators in the single-mediator model at the 6-month follow-up (Figure 2). The model with multiple mediators included direct paths from all mediators to the outcome (OC) and from FM and SC to ER. We fitted models in the structural equation model (SEM) framework in Stata 16 (28), using full information maximum likelihood and conditioning on covariates to account for missing data under the missing at random assumption.

We adjusted at baseline for the mediators and outcome values for the following covariates in the models: sex, age, educational level, years as informal caretaker, schizophrenia, and anxiety (diagnosis of loved one). We allowed for correlations between baseline mediator and outcome measurements errors in order to prevent contamination of the b paths and allowed for correlations of the errors of the mediator and outcome measurements over time. In the model with multiple mediators, we allowed for contemporaneous correlations between the errors of the mediator measurements.

In order to estimate the mediated effect of CCT on outcomes at 6 months in the model with single mediators, we first identified all paths that went from randomization to outcome at 6 months through any measure of the mediator and for each type of mediator. We multiplied the coefficients within each of these paths and added the path specific products to obtain the mediated effect. In the model with multiple mediators, there were five types of mediated effects: mediated, only by FM, only by SC, only by ER, by FM and ER, and by SC and ER. For each of these, we identified the relevant path and proceeded as above. The unmediated effect was estimated based on paths from randomization to outcome at 6 months not passing a mediator (all paths from randomization going to outcomes at 6 months that started with a direct path for randomization to outcome at 2 months). The total effect was the combined effect *via* all paths from randomization to outcome (the sum of all the mediated effects and the unmediated effect).

We estimated the 95% CIs of the results of the overall a paths, the mediated, the unmediated, and the total effect, by use of 50 bootstrap replications. The goodness of fit of the models was tested by the chi-squared test, the comparative fit index (CFI), and the root mean squared error of approximation (RMSEA). We used the following criteria to evaluate model fit; a CFI above 0.90 indicates a good model fit. An RMSEA below 0.08 indicates an acceptable model fit, and an RMSEA below 0.05 indicates a good model fit (27–29).

## Results

### DASS-Depression

Results of the single mediation model showed that SC, FM, and ER all had a statistically significant overall mediated effect for CCT on depression at the 6-month follow-up (Supplementary Table 1). Results of the model with ES as a single mediator did not show statistically significant mediated effects (Supplementary Table 1).

Results of the multiple-mediator model including SC, FM, and ER (Figure 2) showed that only SC and FM remained statistically significant mediators for the effect of CCT on symptoms for depression at 6 months: SC: −1.81 (95% CI −3.31 to −0.31) and FM: −1.98 (95% CI −3.65 to 0.33) (Table 2). The mediated effect of ER attenuated and was statistically insignificant: −0.14 (95% CI −1.31 to 1.02). Results suggested that both the overall a path and the b path were statistically insignificant when adjusted for the mediated effects of SC and FM (Table 2). Moreover, the mediated effects from SC and FM through ER were statistically insignificant: SC -> ER: 0.05 (95% CI −0.32 to 0.41); FM -> ER: 0.00 (95% CI −0.16 to 0.17) (Table 2). Goodness-of-fit tests in terms of RMSEA and CFI suggested acceptable model fits (Table 2).

**Table 2:** Mediated, unmediated, and total effects for compassion cultivating training (CCT) on symptoms of depression, anxiety, and stress (DASS) in caregivers of people with mental illness at the 6-month follow-up in a longitudinal path model design with four repeated measurements in an RCT (*n* = 161)^a^.

		**Depression**	**Anxiety**	**Stress**
		Estimate (95% CI) *p*-value^b^	Estimate (95% CI) *p*-value^b^	Estimate (95% CI) *p*-value^b^
**Path coefficients**				
a paths				
a^1^	CCT->SC_2_	5.49 (3.54–7.44) <0.001	5.43 (3.47 to 7.40) <0.001	5.50 (3.54 to 7.45) <0.001
a^2^	CCT->SC_3_	1.29 (−0.95 to 3.54) 0.26	1.36 (−0.88 to 3.60) 0.23	1.33 (−0.90 to 3.56) 0.24
a^3^	CCT->SC_6_	−1.40 (−3.62 to 0.82) 0.22	−1.42 (−3.64 to 0.81) 0.21	−1.41 (−3.64 to 0.82) 0.21
Overall a	CCT->->SC_6_	5.18 (3.20 to 7.16) <0.001	5.16 (3.18 to 7.14) <0.001	5.21 (3.25 to 7.17) <0.001
a^1^	CCT->FM_2_	4.39 (2.59 to 6.18) <0.001	4.34 (2.54 to 6.14) <0.001	4.34 (2.55 to 6.13) <0.001
a^2^	CCT->FM_3_	−0.77 (−2.60 to 1.06) 0.41	−0.76 (−2.60 to 1.07) 0.42	−0.79 (−2.62 to 1.05) 0.40
a^3^	CCT->FM_6_	1.21 (−0.62 to 3.03) 0.19	1.22 (−0.61 to 3.04) 0.19	1.22 (−0.61 to 3.04) 0.19
Overall a	CCT->->FM_6_	4.71 (2.79 to 6.63) <0.001	4.68 (2.71 to 6.64) <0.001	4.68 (2.77 to 6.58) <0.001
a^1^	CCT->ER_2_	5.00 (3.07 to 6.94) <0.001	5.02 (3.07 to 6.97) <0.001	5.05 (3.11 to 7.00) <0.001
a^2^	CCT->ER_3_	−1.48 (−3.66 to 0.70) 0.18	−1.46 (−3.66 to 0.74) 0.19	−1.50 (−3.70 to 0.69) 0.18
a^3^	CCT->ER_6_	0.36 (−2.69 to 1.98) 0.77	−0.38 (−2.73 to 1.97) 0.75	−0.38 (−2.72 to 1.97) 0.75
Overall a	CCT->->ER_6_	4.36 (−0.50 to 9.22) 0.08	4.47 (−0.12 to 9.07) 0.06	4.45 (−0.87 to 9.76) 0.10
Mediator to mediator (constrained)				
	SC->ER	−0.11 (−0.22 to 0.01) 0.07	0.11 (−0.23 to 0.01) 0.07	−0.11 (−0.23 to 0.01) 0.07
	FM->ER	−0.00 (0.10 to 0.09) 0.93	−0.01 (−0.11 to 0.09) 0.87	−0.01 (−0.10 to 0.09) 0.92
b paths (constrained)				
	SC->OC	−0.15 (−0.26 to −0.04) 0.01	−0.05 (−0.12 to 0.03) 0.21	−0.15 (−0.27 to −0.04) 0.01
	FM->OC	−0.22 (−0.35 to −0.10) <0.001	−0.12 (−0.20 to −0.03) 0.01	−0.30 (−0.44 to −0.16) <0.001
	ER->OC	−0.02 (−0.13 to 0.09) 0.79	0.02 (−0.06 to 0.09) 0.68	−0.04 (−0.15 to 0.08) 0.55
**Effects**				
Mediated				
	CCT->SC->OC (all paths including ≥1 SC and not *via* ER)	−1.81 (−3.31 to −0.31) 0.02	−0.71 (−1.82 to 0.40) 0.21	−1.44 (−2.84 to −0.05) 0.04
	CCT->FM->OC (all paths including ≥1 FM and not *via* ER)	−1.98 (−3.65 to −0.33) 0.02	−1.24 (−2.39 to −0.09) 0.04	−2.17 (−3.63 to −0.71) <0.001
	CCT->ER->OC (all paths including ≥1 ER)	−0.14 (−1.31 to 1.02) 0.81	0.18 (−1.04 to 1.40) 0.77	−0.27 (−1.51 to 0.98) 0.64
	CCT->SC->ER->OC (all paths including ≥1 SC and 1 ER)	0.05 (−0.32 to 0.41) 0.80	−0.06 (−0.45 to 0.34) 0.78	0.10 (−0.38 to 0.58) 0.69
	CCT->FM->ER->OC (all paths including ≥1 FM and 1 ER)	0.00 (−0.16 to 0.17) 0.99	−0.00 (−0.19 to 0.18) 0.97	0.00 (−0.21 to 0.22) 0.97
Unmediated	CCT->OR (all paths not *via* SC, FM or ER)	0.43 (−2.25 to 3.10) 0.76	0.44 (−1.69 to 2.58) 0.68	−0.03 (−3.20 to 3.14) 0.99
Total		−3.47 (−6.00 to −0.96) 0.01	−1.38 (−3.36 to 0.60) 0.17	−3.81 (−6.86 to −0.75) 0.02
**Goodness of fit**				
(Degrees of freedom) = X^2^, *p*		(188) = 269, <0.001	(188) = 247.3, <0.001	(188) = 262, <0.001
RMSEA		0.07	0.06	0.07
CFI		0.94	0.95	0.94
Successful bootstrap out of 50		50	45	50

*At baseline residual covariance between all mediators and outcome. At each follow-up time, residual covariance between all mediators. For each mediator and the outcome residual covariance over time*.

a *According to Figure 2*.

b *Adjusted for sex, age, educational level, years as informal caretaker, schizophrenia (diagnosis, loved ones), anxiety (diagnosis, loved ones)*.

*SC, Self-Compassion Scale-12, Neff; FM, Five-Facet Mindfulness Questionnaire-15; ER, Cognitive Reappraisal, The Emotion Regulation Questionnaire; OC, outcome*.

### DASS-Anxiety

All the models with symptoms of anxiety as outcome did not show statistically significant total effects (Table 2; Supplementary Table 1). However, the single model with FM, SC, and ER as mediators suggested statistically significant a and b paths and thereby also statistically significant mediated effects. The model with ES as a single mediator did not show either statistically significant a or b paths (Supplementary Table 1). Results of the multiple-mediator model including SC, FM, and ER (Figure 2) suggested the solely mediated effect of FM for CCT on symptoms on anxiety at the 6-month follow-up: FM: −0.12 (95% CI −0.20 to −0.03) (Table 2). Goodness-of-fit tests in terms of RMSEA and CFI suggested acceptable model fits (Table 2).

### DASS-Stress

Lastly, the single model with SC, FM, and ER as mediators showed statistically significant mediated effects on symptoms of stress, and the total effects were statistically significant (Supplementary Table 1). Results of ES in the single-mediator model suggested that the mediated effect of ES for CCT on symptoms of stress at 6 months of follow-up was statistically insignificant (Supplementary Table 1).

Results of the multiple-mediator model including SC, FM, and ER (Figure 2) showed that the statistically significant mediated effects of SC and FM remained: SC: −1.44 (95% CI −2.83 to −0.05); FM: −2.17 (95% CI −3.63 to −0.71). However, the mediated effect of ER for CCT on stress attenuated and was statistically insignificant: −0.27 (95% CI −1.51 to 0.98). The results showed that both the overall a path and the b path estimate attenuated when adjusted for the mediated effects of self-compassion and mindfulness (Table 2). Goodness-of-fit tests in terms of RMSEA and CFI suggested acceptable model fits (Table 2).

## Discussion

We found strong indications for mindfulness and self-compassion as mediators of the effect of CCT on symptoms of depression and stress at the 6-month follow-up in caregivers of people with mental illness. Regarding symptoms of anxiety, we only found indication for mindfulness as the mediator. We found no support for our hypothesis regarding emotion regulation (ER and ES) as mediators, and mediation of emotion regulation through mindfulness or self-compassion was also not found.

A systematic review (30) investigated the mechanisms of change in the relationship between self-compassion, emotion regulation, and mental health (30). All studies observed a significant negative relationship between self-compassion and emotion dysregulation. The findings were suggested to support the hypothesis that self-compassion works through emotion regulation to influence mental health outcomes. Limitations of the studies included small sample size and cross-sectional designs, thereby not obtaining temporal sequencing (30).

Contrary to these findings, our study suggests that cognitive emotion regulation strategies are not mediating effects in decreasing psychological distress and that self-compassion and mindfulness are. Taken together, these results may suggest that self-compassion and mindfulness are not cognitive emotion-regulating strategies but may be thought of as self-regulating or implicit emotion regulating strategies (30).

Vago and Silbersweig (31) proposed a complex theory of the mechanisms of mind training. The framework focuses on self-processing and the underlying neural systems involved in self-awareness, -regulation, and -transcendence (S-ART). According to S-ART, perceptions, cognitions, and emotions related to our daily ordinary experiences may be biased, leading to unhealthy habits of mind with or without psychopathology (31). Mind training, including the practice of mindfulness and compassion, leads to the development of 1) self-awareness, 2) self-regulation, and 3) self-transcendence. Self-processing and self-regulation may therefore be heavily influenced through mind training and social interactions.

In the light of the above framework, caregivers are motivated to learn skills to take better care of their own mental health, with an intention to do so by completing an intervention. During the intervention, they practice increasing and regulating attention and emotions toward kindness and compassion. This leads to a shift from external to internal awareness, and experiencing the benefits of mindfulness and compassion training from the inside. The educational component of compassion throughout the course helps caregivers understand that suffering is universal. Acknowledging and holding the suffering in a mindful and self-compassionate atmosphere may lead to the reduced symptoms of stress, anxiety, and depression. Furthermore, while biased cognition often directs a person to isolation, blame, and judgment, being in a group offers an opportunity for kindness and connection toward self and others. Therefore, as emotions are co-regulated through interactions, we suggest that learning in a group may also impact the caregivers' ability to reduce suffering.

### Strengths and Limitations

This is the first study to date that has investigated multiple mediators of the manualized CCT program, on symptoms of depression, anxiety, and stress. We investigated four potential mediators, established a timeline with four time points, and used a theory as an underlying guide. In addition, we analyzed the mediated and unmediated effects simultaneous by use of longitudinal path models in an RCT design inspired by Goldsmith et al. (27). Including multiple mediators highlights the importance of adjusting for the effect of the most promising mediators. When analyzed in single mediation models, cognitive reappraisal seemed to be a statistically significant mediator. However, when adjusted for the mediating effects of mindfulness and self-compassion, the effect disappeared.

We assumed the relationship between mediator and outcome (conceptual theory) to be reasonably consistent over time. Hence, we made the b paths equal as constant b paths should provide a more precise estimation (27). We choose to use contemporaneous b paths (32), because we expected that changes in the mediators, as well as the outcomes, began during the CCT intervention, occurring before the first post randomization measurement (26). Our results of the a paths and previously published total effects (12) also showed that the CCT effects occurred primarily at the first post measurement point but remained at the 6-month follow-up (12) (Table 2, except for ER).

We did not find statistically significant total effects on anxiety at 6 months (Table 2). In our previously published effect analysis, we found a statistically significant effect of CCT on anxiety at the 6-month follow up: −2.12 (95% CI −3.96 to −0.29) (12). The difference in results may be explained by use of different statistical models. Our previous analysis was conducted using a repeated-measurement mixed-effect model according to protocol (12). The purpose of the current path analysis was to investigate mediation. Kraemer et al. (32) suggest that it makes sense to investigate mediating effects even without statistically significant total effects.

Unfortunately, it was not feasible to adjust for measurement errors of latent variables as suggested by Goldsmith et al. (27). As they only illustrated the application of the model on simulated data, their tutorials were not compatible with real data (27). Hence, it is a limitation that our models only include observed measurements, thereby including measurement errors. However, our analysis addressed the limitations described in previous systematic reviews and meta-analysis (6, 9, 10) and followed the recommendations of Kazdin for research on mediators (6, 11).

## Conclusion

Mindfulness and self-compassion are important components in reducing the psychological distress experienced by caregivers of people with a mental illness. These results contribute to the knowledge about the underlying mechanisms of a CCT program.

## Data Availability Statement

The original contributions presented in the study are included in the article/Supplementary Material, further inquiries can be directed to the corresponding author/s.

## Ethics Statement

The studies involving human participants were reviewed and approved by Central Denmark Region Committee of Health Research Ethics (De Videnskabsetiske Komitéer for Region Midtjylland) with approval 238/2017. The patients/participants provided their written informed consent to participate in this study.

## Author Contributions

NH, LF, MF, and LJ developed the study concept and study design. Data collection was performed by NH, LJ, and MF performed the data analysis and interpretation. NH drafted the manuscript and LJ and LF provided critical revisions. All authors approved the final version of the manuscript for submission.

## Funding

This work was partially supported by grants from Trygfonden (LJ: ID: 117789) and the Novo Nordisk Foundation (LJ: ID: NNF15OC0018140).

## Conflict of Interest

The authors declare that the research was conducted in the absence of any commercial or financial relationships that could be construed as a potential conflict of interest.

## Publisher's Note

All claims expressed in this article are solely those of the authors and do not necessarily represent those of their affiliated organizations, or those of the publisher, the editors and the reviewers. Any product that may be evaluated in this article, or claim that may be made by its manufacturer, is not guaranteed or endorsed by the publisher.

## Supplementary Material

Supplementary Table 1 Results of single mediater path models with four repeated measurements in an RCT: Mediated, Unmediated, and Total effects for Compassion Cultivating Training (CCT) on symptoms of Depression, Anxiety and Stress (DASS) in caregivers of people with mental illness at 6-months follow-up (*n* = 161)^a^.
