# Supplementary material for: Mediators for the Effect of Compassion Cultivating Training: A Longitudinal Path Analysis in a Randomized Controlled Trial Among Caregivers of People With Mental Illness
Source: Front Psychiatry. 2021 Dec 7;12:761806. doi: 10.3389/fpsyt.2021.761806 (PMC8688838; doi:10.3389/fpsyt.2021.761806)
Supplement: Supplementary Table 1 — Results of single mediater path models with four repeated measurements in an RCT: Mediated, Unmediated, and Total effects for Compassion Cultivating Training (CCT) on symptoms of Depression, Anxiety and Stress (DASS) in caregivers of people with mental illness at 6-months follow-up (n = 161)a. [file Table_1.docx]

**Supplemental Table 1.** Results of single mediater path models with four repeated measurements in an RCT: Mediated, Unmediated and Total effects for Compassion Cultivating Training (CCT) on symptoms of Depression, Anxiety and Stress (DASS) in Caregivers of People With Mental Illness at 6 -Months Follow-up (n=161)^a^

|  |  | **Mediater** | | | |
| --- | --- | --- | --- | --- | --- |
|  |  | **Self-Compassion Scale-12, Neff (SC)**  estimate (95% CI) p-value^b^ | **Five Facet Mindfulness Questionnaire-15 (FM)**  estimate (95% CI) p-value^b^ | **Cognitive Reappraisal, The Emotion Regulation Questionnaire (ER)**  estimate (95% CI) p-value^b^ | **Expressive Suppression, The Emotion Regulation Questionnaire (ES)**  estimate (95% CI) p-value^b,c^ |
| **Outcome (OC)** |  |  |  |  |  |
| **Depression** |  |  |  |  |  |
| **Path coefficients** |  |  |  |  |  |
| *a path* |  |  |  |  |  |
| a^1^ | CCT->mediator_2_ | 5.10 (3.15 to 7.06) <0.001 | 4.65 (2.81 to 6.49) <0.001 | 4.56 (2.78 to 6.34) <0.001 | -1.22 (-2.46 to 0.02) 0.06 |
| a^2^ | CCT->mediator_3_ | 1.86 (-0.52 to 4.23) 0.13 | -1.09 (-3.08 to 0.90) 0.29 | -1.37 (-3.41 to 0.67) 0.19 | -0.27 (-1.47 to 0.94) 0.66 |
| a^3^ | CCT->mediator_6_ | -1.79 (-4.18 to 0.59) 0.14 | 1.45 (-0.38 to 3.29) 0.12 | -0.73 (-3.01 to 1.55) 0.53 | -0.49 (-1.78 to 0.81) 0.46 |
| overall a | CCT->mediator_6_ | 4.90 (2.77 to 7.03) <0.001 | 5.05 (3.20 to 6.91) <0.001 | 2.42 (0.43 to 4.40) 0.02 | -1.13 (-2.68 to 0.42) 0.15 |
| *b path* | Mediator->OC | -0.26 (-0.36 to-0.16) <0.001 | -0.34 (-0.45 to -0.23) <0.001 | -0.21 (-0.31 to -0.12) <0.001 | 0.24 (0.10 to 0.38) <0.001 |
| (constrained) |  |  |  |  |  |
| **Effects** |  |  |  |  |  |
| Mediated | CCT->mediator->OC (all paths including ≥ 1 mediator) | -3.30 (-5.70 to -0.90) <0.001 | -3.41 (-4.89 to -1.93) <0.001 | -1.74 (-2.92 to -0.56) <0.001 | -0.59 (-1.34 to 0.16) 0.12 |
| Unmediated | CCT->OC (all paths not via mediator) | 0.03 (-3.17 to 3.22) 0.99 | -0.46 (-3.19 to 2.27) 0.74 | -1.86 (-4.09 to 0.38) 0.10 | -3.80 (-6.91 to -0.69) 0.02 |
| Total |  | -3.27 (-5.66 to -0.88) 0.01 | -3.87 (-6.34 to -1.40) <0.001 | -3.60 (-5.85 to -1.35) <0.001 | -4.39 (-7.56 to -1.22) 0.01 |
| **Goodness of fit** |  |  |  |  |  |
| (degrees of freedom)=X^2^, p |  | (70)=70.0, 0.48 | (70) =85.7, 0.10 | (70)=70.4, <0.001 | (34)=78.6, <0.001 |
| RMSEA |  | <0.001 | 0.04 | 0.02 | 0.09 |
| CFI |  | 1.00 | 0.98 | 0.99 | 0.92 |
| Successful bootstrap out of 50 |  | 48 | 50 | 41 | 50 |
| **Anxiety** |  |  |  |  |  |
| **Path coefficients** |  |  |  |  |  |
| *a path* |  |  |  |  |  |
| a^1^ | CCT->mediator_2_ | 5.01 (3.04 to 6.98) <0.001 | 4.78 (2.91 to 6.64) <0.001 | 4.67 (2.89 to 4.46) <0.001 | -1.22 (-2.46 to 0.03) 0.06 |
| a^2^ | CCT->mediator_3_ | 2.09 (-0.26 to 4.45) 0.08 | -1.07 (-3.07 to 0.93) 0.29 | -1.31 (-3.32 to 0.71) 0.21 | -0.28 (-1.48 to 0.93) 0.65 |
| a^3^ | CCT->mediator_6_ | -1.90 (-4.30 0.51) 0.12 | 1.44 (-0.40 to 3.27) 0.13 | -0.75 (-3.03 to 1.53) 0.52 | -0.49 (-1.78 to 0.81) 0.46 |
| overall a | CCT->mediator_6_ | 4.90 (2.85 to 6.95) <0.001 | 5.20 (3.04 to 7.35) <0.001 | 2.53 (0.57 to 4.49) 0.01 | -1.13 (-2.67 to 0.41) 0.15 |
| *b path* | Mediator->OC | -0.10 (-0.16 to -0.04) <0.001 | -0.14 (-0.21 to -0.08) <0.001 | -0.09 (-0.15 to -0.03) <0.001 | 0.05 (-0.04 to 0.14) 0.28 |
| (constrained) |  |  |  |  |  |
| **Effects** |  |  |  |  |  |
| Mediated | CCT->mediator->OC (all paths including ≥ 1 mediator) | -1.48 (-2.53 to -0.41) 0.01 | -1.74 (-2.73 to -0.73) <0.001 | -0.84 (-1.48 to -0.19) 0.01 | -0.14 (-0.44 to 0.16) 0.37 |
| Unmediated | CCT->OC (all paths not via mediator) | 0.26 (-1.94 to 2.46) 0.82 | 0.17 (-1.94 to 2.28) 0.88 | -0.77 (-2.93 to 1.38) 0.48 | -1.39 (-3.12 to 0.33) 0.11 |
| Total |  | -1.21 (-3.09 to 0.68) 0.21 | -1.56 (-3.59 to 0.47) 0.13 | -1.61 (-3.70 to 0.48) 0.13 | -1.53 (-3.29 to 0.22) 0.09 |
| **Goodness of fit** |  |  |  |  |  |
| (degrees of freedom)=X^2^, p |  | (70)=72.4, <0.001 | (70)=83.0, <0.001 | (70)=58.9, <0.001 | (41)=63.7, 0.01 |
| RMSEA |  | 0.02 | 0.04 | <0.001 | 0.07 |
| CFI |  | 1.00 | 0.98 | 1.00 | 0.94 |
| Successful bootstrap out of 50 |  | 42 | 48 | 0 | 50 |
| **Stress** |  |  |  |  |  |
| **Path coefficients** |  |  |  |  |  |
| *a path* |  |  |  |  |  |
| a^1^ | CCT->mediator_2_ | 5.24 (3.30 to 7.17) <0.001 | 4.61 (2.77 to 6.45) <0.001 | 4.49 (2.73 to 6.26) <0.001 | -1.54 (-2.82 to -0.26) 0.02 |
| a^2^ | CCT->mediator_3_ | 1.94 (-0.41 to 4.29) 0.11 | -1.17 (-3.18 to 0.85) 0.26 | -1.32 (-3.35 to 0.72) 0.20 | 0.04 (-1.32 to 1.41) 0.95 |
| a^3^ | CCT->mediator_6_ | -1.82 (-4.20 to 0.57) 0.14 | 1.46 (-0.37 to 3.30) 0.12 | -0.74 (-3.02 to 1.54) 0.53 | -0.29 (-1.63 to 1.05) 0.67 |
| overall a | CCT->mediator_6_ | 5.06 (2.97 to 7.15) <0.001 | 5.03 (2.90 to 7.16) <0.001 | 2.40 (0.43 to 4.37) 0.02 | -1.58 (-2.90 to -0.25) 0.02 |
| *b path* | Mediator->OC | -0.27 (-0.38 to -0.16) <0.001 | -0.40 (-0.53 to -0.27) <0.001 | -0.21 (-0.32 to -0.10)<0.001 | 0.11 (-0.01 to 0.22) 0.08 |
| (constrained) |  |  |  |  |  |
| **Effects** |  |  |  |  |  |
| Mediated | CCT->mediator->OC (all paths including ≥ 1 mediator) | -3.13 (-4.73 to -1.53) <0.001 | -3.35 (-4.77 to -1.93) <0.001 | -1.53 (-2.64 to -0.41) 0.01 | -0.43 (-0.94 to 0.07) 0.09 |
| Unmediated | CCT->OC (all paths not via mediator) | -0.30 (-3.33 to 2.72) 0.84 | -0.77 (-3.81 to 2.27) 0.62 | -2.31 (-5.11 to 0.50) 0.11 | -3.97 (-6.86 to -1.08) 0.01 |
| Total |  | -3.43 (-6.35 to -0.51) 0.02 | -4.12 (-7.12 to -1.13) 0.01 | -3.83 (-6.70 to -0.96) 0.01 | -4.40 (-7.20 to -1.61) <0.001 |
| **Goodness of fit** |  |  |  |  |  |
| (degrees of freedom)=X^2^, p |  | 70)=72.5, 0.40 | (70) =98.1, 0.02 | (70)=71.9, 0.42 | (70)=58.0, 0.85 |
| RMSEA |  | 0.02 | 0.06 | 0.02 | <0.001 |
| CFI |  | 1.00 | 0.95 | 1.00 | 1.00 |
| Successful bootstrap out of 50 |  | 50 | 50 | 46 | 25 |

At baseline residual covariance between all mediators and outcome. For each mediator and the outcome residual covariance over time (except model with ES and depression and anxiety as outcome).

^a^ According to figure 1. ^b^Adjusted for sex, age, educational level, years as informal caretaker, schizophrenia (diagnosis, loved ones), anxiety (diagnosis, loved ones). ^c^Adjusted for age, sex (in models with depression and anxiety as outcome).

DASS Depression Anxiety Stress Scale

RCT Randomized Controlled Trial
